# Evaluation of the Feasibility of Using Commercial Wound Coatings as a Carrier Matrix for Bacteriophages

**DOI:** 10.17691/stm2024.16.1.05

**Published:** 2024-02-28

**Authors:** V.V. Beschastnov, I.Yu. Shirokova, N.A. Belyanina, I.E. Pogodin, A.A. Tulupov, A.G. Tochilina, I.V. Belova, Yu.O. Tyumenkov, O.V. Kovalishena, I.V. Soloveva

**Affiliations:** Professor, Senior Researcher, University Clinic; Privolzhsky Research Medical University, 10/1 Minin and Pozharsky Square, Nizhny Novgorod, 603005, Russia; Head of the Bacteriology Laboratory, Research Institute of Preventive Medicine; Privolzhsky Research Medical University, 10/1 Minin and Pozharsky Square, Nizhny Novgorod, 603005, Russia; Associate Professor, Department of Epidemiology, Microbiology and Evidence-Based Medicine; Privolzhsky Research Medical University, 10/1 Minin and Pozharsky Square, Nizhny Novgorod, 603005, Russia; Junior Researcher, University Clinic; Privolzhsky Research Medical University, 10/1 Minin and Pozharsky Square, Nizhny Novgorod, 603005, Russia; Orthopedic Traumatologist, Head of the Burns Department (for Adults), University Clinic; Privolzhsky Research Medical University, 10/1 Minin and Pozharsky Square, Nizhny Novgorod, 603005, Russia; Junior Researcher, University Clinic; Privolzhsky Research Medical University, 10/1 Minin and Pozharsky Square, Nizhny Novgorod, 603005, Russia; Associate Professor, Department of Epidemiology, Microbiology and Evidence-Based Medicine; Privolzhsky Research Medical University, 10/1 Minin and Pozharsky Square, Nizhny Novgorod, 603005, Russia; Senior Researcher, Laboratory of Human Microbiome and Means for its Correction; Academician I.N. Blokhina Nizhny Novgorod Scientific Research Institute of Epidemiology and Microbiology of Rospotrebnadzor (Russian Federal Consumer Rights Protection and Human Health Control Service), 71 Malaya Yamskaya St., Nizhny Novgorod, 603950, R; Associate Professor, Department of Epidemiology, Microbiology and Evidence-Based Medicine; Privolzhsky Research Medical University, 10/1 Minin and Pozharsky Square, Nizhny Novgorod, 603005, Russia; Leading Researcher, Laboratory of Human Microbiome and Means for its Correction; Academician I.N. Blokhina Nizhny Novgorod Scientific Research Institute of Epidemiology and Microbiology of Rospotrebnadzor (Russian Federal Consumer Rights Protection and Human Health Control Service), 71 Malaya Yamskaya St., Nizhny Novgorod, 603950, R; Junior Researcher, University Clinic; Privolzhsky Research Medical University, 10/1 Minin and Pozharsky Square, Nizhny Novgorod, 603005, Russia; Professor, Head of the Department of Epidemiology, Microbiology and Evidence-Based Medicine; Privolzhsky Research Medical University, 10/1 Minin and Pozharsky Square, Nizhny Novgorod, 603005, Russia; Director of the Research Institute of Preventive Medicine, University Clinic; Privolzhsky Research Medical University, 10/1 Minin and Pozharsky Square, Nizhny Novgorod, 603005, Russia; Associate Professor, Leading Researcher, Head of the Laboratory of Human Microbiome and Means for its Correction; Academician I.N. Blokhina Nizhny Novgorod Scientific Research Institute of Epidemiology and Microbiology of Rospotrebnadzor (Russian Federal Consumer Rights Protection and Human Health Control Service), 71 Malaya Yamskaya St., Nizhny Novgorod, 603950, Russia

**Keywords:** commercial wound dressings, infected wound, carrier matrix, bacteriophage, antibiotic resistance of *Staphylococcus aureus*, MRSA

## Abstract

**The aim of the investigation** is to study the possibility of applying commercial wound coatings for treating infected wounds as a carrier matrix for bacteriophages.

**Materials and Methods:**

Twelve varieties of commercial wound coverings based on biopolymers of natural and synthetic origin, a biological preparation Staphylophag produced by scientific-industrial association Microgen (Russia), registration certificate P N001973/01, and the *S. aureus* 3196 test strain (GenBank JARQZO000000000) isolated from a patient with a burn wound have been used in our work. The ability of commercial biological wound coatings to absorb solutions was examined by immersing them in a physiological solution (pH 7.0−7.2) followed by weighing. The lytic activity of three bacteriophage series against the test strain was studied using the Appelman method and a spot test. The lytic activity of the bacteriophage in the wound samples was studied within 7 days after its absorption by the wound coatings.

**Results:**

The greatest volume of fluid was absorbed by the LycoSorb, NEOFIX FibroSorb Ag, Biatravm, and Chitocol-S wound coatings. All bacteriophage series have been found to have a high lytic activity against the test strain. It has also been shown that Chitocol-S, Collachit-FA, Algipran, and Aquacel Ag Extra possessed their own inherent antibacterial activity under *in vitro* conditions stable for 7 days; moreover, the lysis zones of the test strain increased after their saturation with bacteriophage. On day 0, a high level of bacteriophage lytic activity with the maximum size of the test strain lysis zones from 49 to 59 mm have been found to remain in all samples of the wound coverings. The bacteriophage activity persisted for 1 day in the samples of Hydrofilm, Polypran, and NEOFIX FibroCold Ag coatings, up to 4 days in Algipran, Nano-Aseptica, and Biatravm coatings; and for 7 days in the Chitocol-S, Collachit-FA, Opsite Post-Op Visible, NEOFIX FibroSorb Ag, Aquacel Ag Extra, and LycoSorb samples.

**Conclusion:**

Modern commercial wound dressings based on chitosan-collagen complex (Chitocol-S, Collachit-FA), polyurethane (Opsite Post-Op Visible, LycoSorb, NEOFIX FibroSorb Ag), and Hydrofiber (Aquacel Ag Extra) have a sufficient level of bacteriophage solution absorption, provide a stable preservation of the bacteriophage lytic activity under *in vitro* conditions up to 7 days. Thus, the *in vitro* studies prove the possibility of their use as a carrier matrix for bacteriophages.

## Introduction

Chronic and long-term wounds, including burns, are characterized by the possibility of being infected with antibiotic-resistant microflora, which makes it difficult to close them with auto-, allo-, or heteroplastic material and is an absolute contraindication to using cell technologies or other products of tissue engineering for their treatment. Antibiotic resistance of microorganisms colonizing wound defects is a global problem stimulating the development of alternative and supplemental methods of antimicrobial therapy. Application of virulent bacteriophages, i.e. viruses causing lysis (destruction) of bacteria, is recognized to be a promising method of struggling against resistant causative agents of wound infection [1–3].

In Russia, application of bacteriophages is provided by “The national concept of preventing health care-associated infections” approved by the Chief State Sanitary Physician in 2011, while the Government strategy in preventing the spreading of antimicrobial resistance for the period up to 2030 implies the development of regulations for the application of bacteriophages in different healthcare fields [4].

A great interest in bacteriophages is determined by their properties: owing to a strict specificity of action, curative and preventive bacteriophages do not inhibit normal microbiota of various human body loci, do not suppress immune defense of a macroorganism, are not toxic, and are equally active against sensitive and antibiotic-resistant strains of microorganisms.

A high specificity and efficacy of bacteriophages is provided by their binding to specific receptors of the host cell causing the destruction of the bacterial cell, which is followed by the yield of mature phage particles capable of infecting new bacterial cells. This makes it possible to use bacteriophages as an alternative means of fighting the agents of wound infection as a monotherapy or in combination with antibiotics, which enables effective prevention of antibiotic-resistant strain formation, including MRSA (methicillin-resistant *S. aureus*), one of the main causative agents of the burn wound infections [5, 6].

The current curative and preventive bacteriophages represent a complex of especially selected, highly virulent bacterial viruses against the most common groups of bacterial infectious agents. For example, a highly specific biological preparation Staphylophag (Microgen, Russia) active against antibiotic-resistant bacteria of the genus *Staphylococcus* acts as a biological agent.

The greatest effectiveness of the biological method of treatment can be achieved using bacteriophages included into the carrier matrix, which is determined by the well-known stages of the wound healing process. Local phagotherapy is effective if principles developed in recent years on the basis of the experimental and clinical studies are followed strictly. One of such principles is creation the phage concentration exceeding some threshold value in the zone of interest. This question is being actively discussed and according to various authors [7–9] this threshold is equal to 10^8^–10^9^ plaque-forming units (PFU/ml) or the concentration exceeding that of the target bacteria by 2 orders of magnitude, that is by 100 times. Presently, the possibility of using up-to-date polymers as carrier matrices to maintain the necessary concentration of phages in the wound defect area is being studied [10, 11].

To widen the scope of bacteriophage application for wound care, it is reasonable to use modern commercial dressings based on natural polymers (chitosan, sodium alginate, cellulose, etc.) and synthetic materials (polyvinyl alcohol, polyurethanes, etc.) as their carrier matrices.

Chitosan is a chitin-derived biodegradable biopolymer consisting of poly-N-acetylglucosamine, widely abundant material in nature, the main component of the marine arthropod exoskeleton. Chitosan is known to possess hemostatic, bactericidal, fungicidal, antioxidant, and wound-healing properties, is shown to be used as a scaffold and drug delivery carrier [12–17]. Sodium alginate is a natural polysaccharide obtained from marine brown and red algae growing in the Indonesian region. It is capable of absorbing the fluid mass 300 times exceeding that of its own [18].

Polyvinyl alcohol represents a synthetic polymer with high biocompatibility, good hydrophilic properties, and biomechanical characteristics. It can easily form hydrogels and is widely used for the production of dressings with controlled drug release. Previously [19], we have received data on a high capacity of the polyvinyl alcohol-based wound coating to liquid absorption, which makes it possible to consider it as a possible bacteriophage carrier matrix. Polyurethane-based wound coatings are also challenging as they are biologically neutral, possess high absorption and retaining capacity, softness, elasticity, and a small mass.

According to the data of the scientific literature [20, 21], all current biopolymers have important properties for wound treatment: they are not toxic, not immunogenic, possess sufficient mechanical strength, and high hydrophilicity. They are capable of effectively absorbing and retaining a large amount of wound exudate and can maintain optimal local moisture conditions [20]. The simplest and most economically beneficial way of bacteriophage immobilization in the area of the wound defect is their absorption by the wound coatings [21].

**The aim of the investigation** is to study the possibility of applying commercial wound coatings for treating infected wounds as a carrier matrix for bacteriophages.

## Materials and Methods

### The applied materials

Commercial wound coatings from different manufacturers for treating patients with longstanding wounds have been selected for the investigation (Table 1).

**Table 1:** Wound coatings used to perform the work

No.	Name	Manufacturer	Description
***Natural polymer-based wound coatings***			
1	Chitocol-S	Evers LLC, Russia	Biodegradable, highly porous, lyophilized sponge based on chitosan and chitosan-collagen complex with inclusion of ultrafine silver particles, anilocain analgesic, and proteolytic enzyme chymotrypsin
2	Algipran	Napoli LLC, Russia	Dressing from nonwoven material — polyacrylonitrile fibers impregnated with sodium alginate solution
3	Collachit-FA	Medical Company “Collachit” LLC, Russia	Biodegradable, lyophilized spongy wound coating based on chitosan and collagen with addition of anilocain and furagin
***Artificial polymer-based wound coatings***			
4	Opsite Post-Op Visible	Smith & Nephew, Great Britain	Moisture-resistant, bacteria-proof polyurethane film with a cellular absorbent honeycomb-like sponge
5	Hydrofilm	Hartmann, Germany	Polyurethane film-based wound dressing
6	NEOFIX FibroSorb Ag	Pharmaplast S.A.E., Egypt	Wound coating containing polyurethane sponge, polyurethane film, silver ions
7	Nano-Aseptica	Nano-Aseptica LLC, Russia	Wound dressing composed of fabric with a silver nanocoating
8	Biatravm	Lintex, Russia	Wound coating based on modified polyester fibers and collagen sponge with antiseptic
9	Polypran	New Dressing Materials LLC, Russia	Hydrophilic film wound coating based on polyvinyl alcohol with lidocaine
10	NEOFIX FibroCold Ag	Pharmaplast S.A.E., Egypt	Hydrocolloid, adhesive wound dressing with silver ions covered from the outside by the antibacterial, water-proof polyurethane film
11	Aquacel Ag Extra	ConvaTec, Great Britain	Hydrofiber-based dressing, which acquires gel consistency coming in contact with the wound exudate
12	LycoSorb	Optimelle, Egypt	Absorbing adhesive dressing impregnated with a combined lipidocolloid composition from paraffin oil, soft paraffin, coupling polymer, hydrocolloid powder, and antioxidant impregnated into foam polyurethane

Staphylophag, further — bacteriophage (Microgen, Russia, registration certificate P N001973/01), active against antibiotic-resistant flora, was used as a model sample. It represents a transparent fluid of yellow color with various intensity and possible greenish tint, whose acting substance is a sterile purified filtrate of genus *Staphylococcus* bacterial phagolysates (with activity of not less than 10^–5^ according to the Appelman method) series H 001, H 003, H 004, and H 286.

A test strain *S. aureus* 3196, isolated from the patient with a burn wound, was comprehensively studied, its biological, molecular, and genetic properties were described. The strain possesses the biochemical properties typical for its genus, lecithinase and hemolytic activity, is referred to the MRSA group, resistant to a wide spectrum of antibiotics: penicillins, cephalosporins, fluoroquinolones, and carbapenems, sensible to tigecycline. Determinants of antibiotic resistance and pathogenicity are presented in the strain genome (Table 2).

**Table 2:** Molecular and genetic properties of the *S. aureus* 3196 test strain

Genome size (bp)	GC-content (%)*	Number of coding sequences	Determinants of antibiotic resistance	Virulence-and pathogenicity-related genes	Sequence-type	Number in the GenBank database
2.771.910	32.9	2.883	*mepR*, *mepA*, *mgrA*, *LmrS*, *norC*, *norA* — molecular efflux pumps *vanT* — resistance to glycopeptides *FosB* — resistance to phosphomycyn *mecA* — resistance to methicillin	*aur* — aureolysin *splA*, *splB*, *splE* — proteases *sak* — staphylokinase *scn* — staphylococcal complement inhibitor *hlgA*, *hlgC* — hemolysins *lukD*, *lukE* — leukocidin D *sea* — enterotoxin A	8	JARQZO000000000

^*^ the percentage of guanine (G) and cytosine (C) among all nucleotide residues of the nucleotide sequence under consideration.

### Investigation of absorbing capacity of wound coatings

A square sample (2×2 cm) was cut with scissors from each type of the wound coating using a prepared stencil and immersed into 0.9% sodium chloride solution (sterile physiological solution), pH 7.0– 7.2. Before immersing into the solution, each sample of the wound coating was weighed on the electronic scale (Acom JW-1-200, Korea). Thereafter the sample was immersed into the solution for 1 min, removed, and 30 s after the fluid has fallen down, was weighed again. Once the result was fixed, the sample was placed in the same solution for 5 min, and weighing was repeated. The results of weighing the 4-cm^2^ samples were then recounted to calculate the fluid mass per 1 cm^2^ of the wound coating surface absorbed after 6 min of being in the solution. The experiment was performed in three repetitions.

### Investigation of Staphylophag activity against the S. aureus 3196 test strain

The activity of different bacteriophage series against the *S. aureus* 3196 test strain was studied using the Appelman method [22] and a spot test [23]. To perform a spot test, a test strain inoculate with the concentration of 1.5**·**10^8^ CFU/ml (0.5 according to the McFarland standard scale) was prepared; then, a solid culture medium (Mueller–Hinton agar, M173; HiMedia, India) was inoculated using a lawn method. A few minutes after the culture has dried, drops of various bacteriophage series in the volume of 30 μl were placed on the surface of the culture medium and incubated at 37±1°C for 24 h. The lysis reactions (the degree of inhibition of the test strain visible growth) were assessed with the naked eye at direct illumination or at an angle of 45° using a 5-point scale [23].

### Investigation of stability of bacteriophage lytic activity in the wound coating samples

The stability of bacteriophage lytic activity in the wound coating samples was studied during a period of performing multiple infected wound dressings (4–7 days). Petri plates with the culture medium (Mueller–Hinton agar) were inoculated with the *S. aureus* 3196 test strain at the concentration of 1.5**·**10^8^ CFU/ml (0.5 McFarland standard scale). Samples of each wound coating type were immersed into the 0.9% sodium chloride solution (control) and bacteriophage (experiment) for 5 min and then transferred under aseptic conditions to the sterile Petri plates. One sample of each wound coating was applied to the Petri plate inoculated with a test culture lawn immediately after saturation with the solutions (day 0 after solution absorption), while the remaining samples were stored in the sterile Petri plates in the thermostat under aseptic conditions and placed on the freshly prepared test strain lawn successively on days 1, 2, 3, 4, and 7 after absorption. Incubation was carried out in the thermostat at 37±1°C for 24 h. The results were assessed in compliance with the guidelines [23]. The experiment was performed in three repetitions.

### Statistical processing

The data obtained were statistically processed using Statistica 10.0 program. Statistical significance of differences between the groups compared by qualitative features was assessed using nonparametric methods: the Wilcoxon test was applied to compare two dependent (related) groups, the Mann–Whitney test was used for comparison of two independent (unrelated) groups. The confidence intervals for relative parameters were evaluated using the Wilson method. Sampling parameters are designated in the following way: Me [Q1; Q3], where Me is the median, Q1 is the upper quartile, Q3 is lower quartile; n is the size of the analyzed subgroup, p is the value of statistically significant differences. The critical value of the significance level was accepted to be equal to 5% (p≤0.05).

## Results

**Figure 1.**
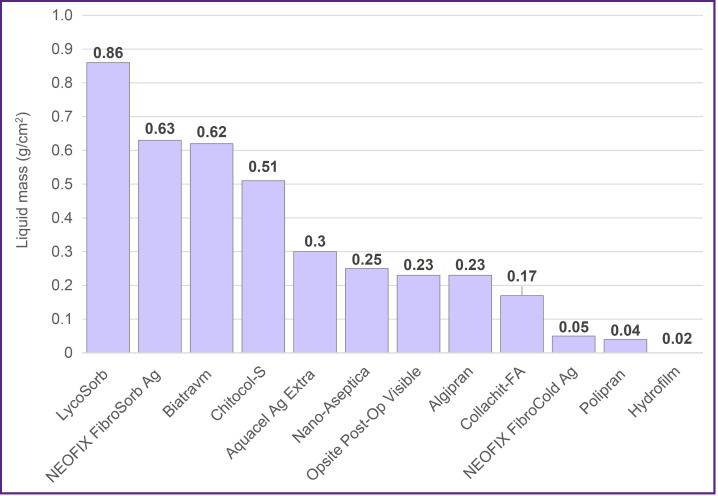
Absorbing capacity of the wound coatings Mass of the physiological solution absorbed by the wound coatings during 6 min (g/cm^2^)

The investigations have shown that samples of the commercial wound coatings possess various capacity of liquid absorption (Figure 1).

A small fluid mass was absorbed by the LycoSorb wound coatings in the volume of 0.86 [0.86; 0.92] g/cm^2^, NEOFIX FibroSorb Ag — 0.63 [0.61; 0.70] g/cm^2^, Biatravm – 0.62 [0.60; 0.67] g/cm^2^, and Chitocol-S — 0.51 [0.48; 0.52] g/cm^2^. In this group, LycoSorb, Chitocol-S, and Biatravm samples were saturated with fluid in 1 min, whereas NEOFIX FibroSorb Ag samples absorbed less than half of the fluid during the first minute, absorbing the remaining part during the next 5 min.

The least volume of fluid was absorbed by the following wound coatings: Hydrofilm — 0.02 [0.01; 0.03] g/cm^2^, Polypran — 0.04 [0.03; 0.04] g/cm^2^, and NEOFIX FibroCold Ag — 0.05 [0.04; 0.05] g/cm^2^; moreover, the entire volume was taken during the first minute and did not actually increase in the subsequent 5 min. The rest samples absorbed the fluid in the volume of 0.17−0.30 g/cm^2^ during the first minute of immersion.

**Figure 2.**
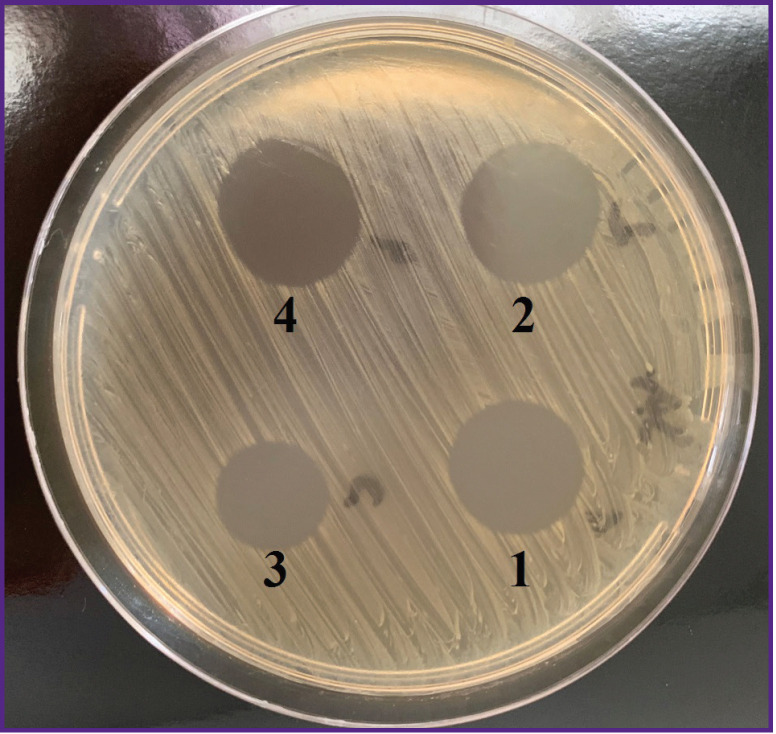
Lytic activity of staphylococcal bacteriophage (Microgen, Russia) against *S. aureus* 3196 test strain: *1* — H 001 series; *2* — H 003 series; *3* — H 004 series; *4* — H 286 series

Investigations of bacteriophage activity using Appelman method have established that all its series provide culture lysis in the titer of at least 10^–5^, while the spot test showed clear zones of culture lysis without any colonies of the secondary growth on the surface of the culture medium at the sites of application of all bacteriophage series (Figure 2). This points to the fact that all bacteriophage series possess a high lytic activity against the *S. aureus* 3196 test strain. A phage series H 286, providing the greatest diameter of the test-strain lysis zone, was chosen for further work.

It has also been established that the wound coatings Chitocol-S, Collachit-FA, Algipran, and Aquacel Ag Extra possess inherent antibacterial activity. On day 0 of absorption, i.e. when samples of these coatings were placed on the test cultures directly after soaking in the 0.9% solution of sodium chloride and incubation for 24 h, a clear lysis zone of the test strain was observed. For the samples of Chitocol-S, Collachit-FA, and Aquacel Ag Extra, the lysis zones were consistently determined during entire 7 days of investigation, the lysis zone for Algipran was noted on days 0, 1, and 2, then on days 3, 4, 7 the lysis zones were absent (Table 3). The rest commercial wound coatings did not show inherent activity against the chosen test strain.

**Table 3:** Zones of lysis of the *S. aureus* 3196 test strain by the bacteriophage series H 286 contained in the commercial wound dressings

Wound coating	Diameter of the lysis zone on different days after absorption (mm)											
	Day 0		Day 1		Day 2		Day 3		Day 4		Day 7	
	NaCl*	Phag	NaCl*	Phag	NaCl*	Phag	NaCl*	Phag	NaCl*	Phag	NaCl*	Phag
Chitocol-S	40	59	40	58	38	55	38	55	35	50	34	49
Algipran	39	55	39	55	35	50	0	49	0	0	0	0
Collachit-FA	38	56	38	56	36	54	36	53	34	52	28	47
Opsite Post-Op Visible	0	55	0	55	0	55	0	53	0	49	0	48
Hydroflm	0	49	0	0	0	0	0	0	0	0	0	0
NEOFIX FibroSorb Ag	0	50	0	50	0	50	0	50	0	47	0	45
Nano-Aseptica	0	49	0	49	0	48	0	44	0	0	0	0
Biatravm	0	51	0	50	0	50	0	46	0	0	0	0
Polypran	0	50	0	0	0	0	0	0	0	0	0	0
NEOFIX FibroCold Ag	0	51	0	0	0	0	0	0	0	0	0	0
Aquacel Ag Extra	38	53	38	53	38	53	37	49	37	48	35	48
LycoSorb	0	58	0	58	0	58	0	54	0	48	0	48

^*^ 0.9% NaCl.

**Figure 3.**
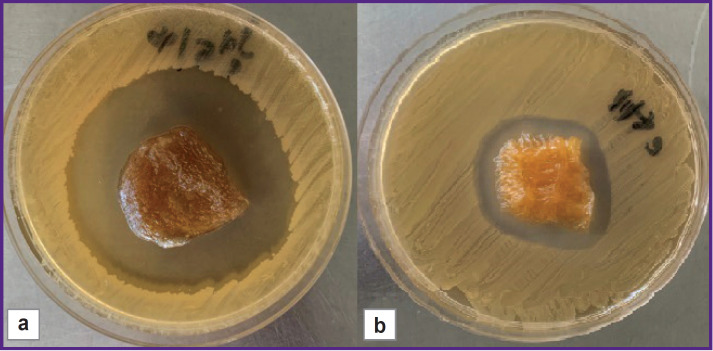
Zones of lysis of the *S. aureus* 3196 test-strain culture exposed to the Chitocol-S wound coating saturated with the bacteriophage (a) and 0.9% sodium chloride solution (b) on day 0 of absorption

All commercial wound coatings, considered as carrier matrices, retained high lytic phage activity on day 0, which characterizes them as biologically neutral towards the employed bacteriophage. The diameter of the lysis zone in the samples possessing inherent antibacterial activity (Chitocol-S, Collachit-FA, Algipran, Aquacel Ag Extra), was 18 [17; 21] mm larger (p<0.001) after the saturation with bacteriophage than the diameter after the saturation with 0.9% sodium chloride solution (Figure 3).

Since the first day of absorption, differences in the lytic activity of the bacteriophage immobilized on various wound coatings were noted. The bacteriophage remained active up to 1 day in the Hydrofilm, Polypran, and NEOFIX FibroCold Ag coatings.

In the Algipran, Nano-Aseptica, Biatravm coatings, the bacteriophage maintained its lytic activity up to 4 days, while in Chitocol-S, Collachit-FA, Opsite Post-Op Visible, NEOFIX FibroSorb Ag, Aquacel Ag Extra, and LycoSorb, the bacteriophage preserved its lytic activity for 7 days. The test-strain lysis zones decreased by the last day of the exposure of the wound coatings absorbing the bacteriophage (see Table 3).

## Discussion

It is necessary to underline that the conducted investigation does not evaluate the quality of the wound dressings, since none of them was developed as a carrier matrix for biological objects and does not have appropriate indications for use. The aim of the investigation was to study the possibility of using these commercial coatings, employed for treating infected burn wounds, as a carrier matrix of bacteriophages, to examine their absorbing capacity and ability to maintain stability of bacteriophage lytic activity during the period necessary for multiple bandages of the infected burn wound, performed on average two times a week under general anesthesia.

Wound coatings are found to take up fluid to a varying extent. The periods of time, 1 and 6 min, for immersing the coatings into the solution were chosen based on clinical appropriateness, as the process of patient preparation, removing a bandage, and exposure of the wound defect take about 5−6 min, and this time may be used for impregnating the wound coating with bacteriophage. The greatest absorbing capacity was shown by the wound coatings designed by the manufactures for treating the wounds in the phase of exudation: LycoSorb, NEOFIX FibroSorb Ag, Biatravm, average capacity was demonstrated by Chitocol-S, Aquacel Ag Extra, Nano-Aseptica, and Collachit-FA. The Hydrofilm coating with minimal absorbing capacity is designed as a secondary bandage for mechanical protection of the wound.

Exploring the duration of viability and lytic capacity of bacteriophage in the wound coatings, it has been found that Chitocol-S, Collachit-FA, and Aquacel Ag Extra possess inherent antibacterial activity under *in vitro* conditions stable during 7 days, moreover, the increased lysis zones of the test strain were observed after their saturation with bacteriophage. The longest lytic bacteriophage activity (up to 7 days) was provided by the Collachit-FA and Chitocol-S coatings based on the chitosan-collagen complex, polyurethane-based coatings Opsite Post-Op Visible, NEOFIX FibroSorb Ag, LycoSorb, and the Hydrofiber-based coating Aquacel Ag Extra.

## Conclusion

Modern commercial wound coverings based on chitosan-collagen complex (Chitocol-S, Collachit-FA), on polyurethane (OpSite Post-Op Visible, LycoSorb, NEOFIX FibroSorb Ag), and on the Hydrofiber (Aquacel Ag Extra) possess a sufficient level of bacteriophage absorption, provide a stable high level of bacteriophage lytic activity under *in vitro* conditions (up to 7 days), i.e. during the period necessary for multiple bandages of the infected burn wound. The *in vitro* results prove the possibility of using these coatings as a carrier matrix for bacteriophages.
